# Plexin-B2 and Semaphorins Do Not Drive Rhabdomyosarcoma Proliferation or Migration

**DOI:** 10.1155/2022/9646909

**Published:** 2022-05-06

**Authors:** Anju Karki, Reshma Purohit, Sofia Tosoni, Narendra Bharathy, Joel E Michalek, Sonja Chen, Charles Keller

**Affiliations:** ^1^Children's Cancer Therapy Development Institute, Beaverton, OR 97005, USA; ^2^Department of Epidemiology and Biostatistics, University of Texas Health Science Center, San Antonio, TX 78229, USA; ^3^Warren Alpert Medical School, Brown University, Providence, RI 02903, USA; ^4^Lifespan Academic Medical Center, Rhode Island Hospital, Providence, RI 02903, USA

## Abstract

Rhabdomyosarcoma (RMS) is the most common pediatric soft tissue sarcoma for which subsets of patients have longstanding unmet clinical needs. For example, children with alveolar rhabdomyosarcoma and metastases at diagnosis will experience only 8% disease-free 5-year survival for nonlocalized unresectable recurrent disease. Hence, development of novel therapeutic strategies is urgently needed to improve outcomes. The Plexin-Semaphorin pathway is largely unexplored for sarcoma research. However, emerging interest in the Plexin-Semaphorin signaling axis in pediatric sarcomas has led to phase I cooperative group dose-finding clinical trials, now completed (NCT03320330). In this study, we specifically investigated the protein expression of transmembrane receptor Plexin-B2 and its cognate SEMA4C ligands in clinical RMS tumors and cell models. By RNA interferences, we assessed the role of Plexin-B2 in cell growth and cell migration ability in selected alveolar and embryonal RMS cell model systems. Our results affirmed expression of Plexin-B2 across human samples, while also dissecting expression of the different protein subunits of Plexin-B2 along with the assessment of preferred Semaphorin ligands of Plexin-B2. Plexin-B2 knockdown had positive or negative effects on cell growth, which varied by cell model system. Migration assayed after Plexin-B2 knockdown revealed selective cell line specific migration inhibition, which was independent of Plexin-B2 expression level. Overall, these findings are suggestive of context-specific and possibly patient-specific (stochastic) role of Plexin-B2 and SEMA4 ligands in RMS.

## 1. Introduction

Rhabdomyosarcoma (RMS) is the most common soft tissue sarcoma in children and accounts for ∼5–10% of all childhood malignancies [1, 2]. Histologically, two major subtypes of RMS are described, alveolar (aRMS) and embryonal (eRMS). The aRMS subtype is known to be clinically aggressive and metastatic in nature with unfavorable prognosis [3, 4]. Despite technical advancement in the treatment strategies (namely, surgery, radiation therapy, and chemotherapy), the survival rate has stayed unchanged for multiple decades [5, 6] with a long-term disease-free survival rate for metastatic aRMS and eRMS of 8% and 43%, respectively [2, 5, 7]. Therefore, development of novel therapeutic strategies is urgently needed to improve cure rates.

Plexins are a family of transmembrane protein receptors that bind to Semaphorin molecules to modulate cellular functions such as cell migration, cell adhesion, and invasion [8, 9]. Specifically, Plexin-B2 is involved in cell motility, vessel formation, and central nervous system development [10–12] by binding to Semaphorin 4C (SEMA4C). Recent finding reported that Plexin-B2-SEMA4C axis also plays a critical role in recruiting T-cells to germinal centers and regulating the antibody responses [13]. Overexpression of Plexin-B2 and SEMA4C has been implicated in the poor prognosis in the cancers of bone, breast, and brain [8, 9, 14–16]. Furthermore, in the context of cancer progression, Plexin-B2-SEMA4C signaling cascade can enhance cell invasion and migration by triggering downstream effector pathways such Met, ErbB2, and RhoA-dependent kinase [17]; Le et al., 2015). In order to better understand the disease metastasis, we investigate the previously unexplored Plexin-Semaphorin signaling pathway in RMS and elucidate the role of this membrane protein complex in the context of disease progression.

Here, we investigated roles for Plexin-B2-SEMA4s in RMS (aRMS and eRMS) disease progression; specifically, we examined the effect of silencing Plexin-B2 on cell growth and migration. In our studies, we have affirmed the expression of Plexin-B2 and its cognate ligands SEMA4C, SEMA4D, and SEMA4F across human samples. Furthermore, we worked carefully to understand the isoforms of Plexin-B2 and the functional significance of Semaphorin ligands that bind to Plexin-B2 using small interfering RNAs of Plexin-B2 in rhabdomyosarcoma cell lines as a genetic proof of concept. Our findings show that cell growth was significantly decreased in both aRMS and eRMS cancer cell lines. Interestingly, silencing of Plexin-B2 enhanced cell growth in a primary aRMS cell culture. However, cell migration capacity was reduced in only one aRMS cell line tested, despite all cell lines expressing Plexin-B2. These results suggest potential patient-to-patient variability for the function of Plexin-Semaphorin signal transduction in RMS that may not be predictable from Plexin-B2 as a biomarker.

## 2. Methods and Materials Cell Culture

Human RMS cell lines, Rh3, Rh5, Rh18, Rh30, and Rh41, were obtained from Childhood Cancer Repository (http://www.cccells.org) and were cultured in RPMI 1640 (Thermo Fisher Scientific (Thermo), Waltham, MA, Cat. #11875-093) containing 10% fetal bovine serum (FBS, Thermo Fisher Scientific, Cat. #26140-079) and 1% penicillin/streptomycin (Thermo, Cat. #15140-122). RD was obtained from ATCC, Manassas, VA (Cat. #CCL-136) and grown in Dulbecco's modified Eagle's media (DMEM) (Thermo, Cat. #11995-065) supplemented with 10% FBS and 1% penicillin/streptomycin. Primary HSMM cell lines were obtained from Lonza Inc., Morristown, NJ (Cat. #CC-2580) and cultured in growth media from Cell Applications, San Diego, CA (Cat. #151-500). SJ-GBM cell line was obtained from Dr. Susan Baker (St. Jude's Hospital) and was grown in a base medium of Iscove's modified Dulbecco's medium (IMDM, Thermo, Cat. #12440061) with 10% FBS, 1% penicillin/streptomycin, 4 mM L-glutamine (Thermo, Cat. #25030081) and 1x insulin, transferrin, and selenous acid (ITS, Corning Life Sciences, Bedford, MA, Cat. #41400045). PDX explant primary cell culture CF-1 was grown in RPMI 1640 containing 10% FBS and 1% penicillin/streptomycin. All cell cultures were maintained at 37°C and 5% CO2. STR analyses of the cell lines are shown in Supplementary Table 1.

### 2.1. siRNA-Mediated Silencing of Plexin-B2

In order to silence Plexin-B2, RD, Rh18, Rh30, Rh41, and CF-1 cells were seeded at 2.5 × 10^5^ density 24 hr prior to transfection. Cells were transfected with 25 picomoles (pmol) and 50 pmol of Plexin-B2 siRNAs or control siRNAs using Lipofectamine 3000 (Thermo, Cat. #L3000001) following the manufacturer's instructions. siRNAs were purchased from Dharmacon (GE Healthcare), and the sequences are as follows: siPLEXINB2 siRNA1 (GCAACAAGCUGCUGUACGC), siplexin-B2 siRNA3 (UGAACACCCUCGUGGCACU), and ON-TARGETplus non-targeting siRNA siControls (D-001810-01). In order to determine the knockdown efficiency, protein lysates were harvested for immunoblot analyses and probed with the following primary antibodies: Plexin-B2 (R&D Systems, Cat. #AF5329; Abcam, Cat. #ab193355; Santa Cruz Biotechnology, Cat. #sc-373969), SEMA4C (Santa Cruz Biotechnology, Cat. #138445), SEMA4D (Abcam, Cat. #ab134128), and SEMA4F (Santa Cruz Biotechnology, Cat. #125264).

### 2.2. Cell Proliferation Assay after Plexin-B2 Silencing

Cell proliferation assay was performed after Plexin-B2 silencing. RMS cells were serum-starved and seeded at 5000/well cell density into 96-well plates (Thermo, Cat. #1360) in triplicate. Cell viability was measured after 24, 48, and 72 hr using the CellTiter-Glo luminescent reagent (Promega, Cat. #G7570) in a BioTek plate reader (Shoreline, WA).

### 2.3. Cell Migration Assay after Plexin-B2 Silencing

In order to determine the effect of Plexin-B2 silencing on cell migration, transwell migration assays were performed using Transwell® permeable supports (Costar, Cat. #3422) with 8 *μ*m polycarbonate membrane. RMS cells, with and without Plexin-B2 knockdown, were serum-starved, resuspended in serum-free media, and seeded in the upper chamber at a density of 5 × 10^4^. The bottom chambers consisted of serum-free media (negative control), with media consisting of 10% FBS (positive control). After 48 hr, cell migration was quantified via colorimetric crystal violet assay. Briefly, after incubation, medium from the inserts was aspirated, which was followed by the removal of non-migrating cells from inner side of the inserts using cotton swab. The migrating cells were fixed in 4% paraformaldehyde and washed in PBS. The cells were then stained with 0.1% crystal violet prepared in 10% methanol. The inserts were washed, air-dried, and subjected to 10% acetic acid to elute the crystal violet dye. The intensity of dye was proportional to the cells migrating to the outer side of inserts. The absorbance of eluted dye was measured at a wavelength of 595 nm using a BioTek plate reader.

### 2.4. Cell Migration Assay Using Real-Time Cell Analysis (RTCA)

The assay was set up on the real-time cell analyzer (xCELLigence RTCA DP, Agilent, Santa Clara, CA) system using CIM-plate 16 (Agilent, Cat. #5665817001). Each test condition was performed in triplicate. RMS cells were serum-starved (16 hr) prior to performing the assay. Wells of the lower chamber of the plate were filled with chemoattractant SEMA4C (R&D Cat. #6125-S4), SEMA4D (R&D Cat. #7470-S4), SEMA4C + SEMA4D (200ng/ml in serum-free media (SFM)), 10% FBS (positive control), and SFM (negative control). The upper chamber was then attached and 50 ul of SFM added to each well. The plate was allowed to equilibrate in the incubator at 37°C and 5% CO2 for 1 hr before taking the baseline cell index (CI) readings for each well. RMS cells (5 × 10^4^ cells/100 ul) were plated into each well of the upper chamber of the plate. The cells were allowed to settle at room temperature for 30 minutes before initiating the assay. Readings were taken every 15 minutes for the duration of the assay (48 hr).

Numerical data expressed as mean ± standard deviation were calculated automatically by the RTCA software package. All CI values were normalized to that of the SFM (negative control).

### 2.5. Histology and Immunohistochemistry (IHC)

Formalin-fixed paraffin-embedded (FFPE) human and mouse RMS tumor tissue microarray (TMA, 4 *μ*m thick) were obtained and prepared in collaboration with the Biopathology Center (Nationwide Children's Hospital, Columbus, OH). Control FFPE tissue array (4 *μ*m thick) consisting of multiple organs from 5 aborted fetus was obtained from US Biolab (Rockville, Maryland). The immunohistochemistry was performed using conventional staining techniques. Tissue sections were deparaffinized in Clearite and rehydrated. TMAs were heat-retrieved in antigen unmasking solution (Vector Laboratories, Cat. #H-3300) for 20 minutes. Next, the tissues were incubated in 3% H2O2 to block the endogenous peroxidase activity followed by 2 hr blocking step using SuperBlock Reagent (Thermo, Cat. #37580) supplemented with Avidin (Vector Laboratories, Cat. #SP-2001). After washing steps, the tissues were probed with primary antibodies plus biotin solution overnight at 4°C. Primary antibodies used are Plexin-B2 (R&D Systems, Cat. #AF5329; Abcam, Cat. #ab193355; Santa Cruz Biotechnology, Cat. #sc-373969), SEMA4C (Santa Cruz Biotechnology, Cat. #138445), SEMA4D (Abcam, Cat. #ab134128), and SEMA4F (Santa Cruz Biotechnology, Cat. #125264). The tissues were incubated in biotinylated secondary antibodies (Abcam, Cat. #ab207995, Cat. #ab6788) for 30 minutes at room temperature followed by 1-hour incubation of the tissues in VECTASTAIN ABC reagent (Vector Laboratories, Cat. #PK-7200) at room temperature. IHC development was performed using ImmPACT diaminobenzidine substrate kit (Vector Laboratories, Cat. #SK-4105) followed by counterstaining using hematoxylin (Vector Laboratories, Cat. #H-3404-100). All images were taken using brightfield microscope. Full digital whole slide scans (40x) are available on request.

### 2.6. TMA IHC Scoring

One pathologist independently evaluated the percentage (0, 1–10%, 11–50%, 51–80%, 81–100%) and intensity (0, no staining: 1+, weak staining: 2+, moderate staining: and 3+, strong staining) of the nucleus, cytoplasm, and membranes of stained tumor cells without knowledge of clinical status.

### 2.7. Protein Extraction and Immunoblotting Analysis

In all experiments, whole cell protein lysates were harvested using ice-cold RIPA buffer (Thermo, Cat. #89900) supplemented with HALT protease/phosphatase inhibitor cocktail (Thermo, Cat. #78441). Protein concentrations were determined using Pierce™ BCA Protein Assay Kit (Thermo, Cat. #23227). Protein samples (20–30 *μ*g) were resolved in 4–20% SDS-PAGE gels (Bio-Rad, Cat. #456–2023) and transferred to PVDF membranes using the Mini Trans-Blot System (Bio-Rad, Cat. #1658030). Membranes were blocked for 2 hr in 5% non-fat dry milk reconstituted in Tris-buffered saline plus 0.1% Tween 20 at room temperature, followed by overnight incubation in primary antibodies at 4°C. Primary antibodies used in this study are Plexin-B2 (R&D Systems, Cat. #AF5329; Abcam, Cat. #ab193355; Santa Cruz Biotechnology, Cat. #sc-373969), SEMA4C (Santa Cruz Biotechnology, Cat. #138445), SEMA4D (Abcam, Cat. #ab134128), SEMA4F (Santa Cruz Biotechnology, Cat. #125264), GAPDH (Cell Signaling Technology, Cat. #2118), and ß-actin (Cell Signaling Technology, Cat. #4790). After 3 × 10 min washes, the membranes were subjected to 1 hr incubation at room temperature in secondary antibodies: anti-mouse (Vector Laboratories, Burlington, CA Cat. #PI-2000) and anti-rabbit (Vector Laboratories, Cat. #PI-1000). Immunoblots were developed and visualized using ECL substrate (Bio-Rad, Cat. #1705061) and an IVIS Lumina II imaging system (Caliper Life Sciences, Hopkinton, MA, USA).

### 2.8. Statistical Analyses

The significance of variation in mean optical density (OD) with treatment was assessed with a linear model of OD in terms of treatment, cell type, and treatment by cell type interaction with a Tukey adjustment to correct for multiple comparisons. All data were analyzed in log units. Statistical testing was two-sided with an experiment-wise significance level of 5%. Results were summarized with the sample size, mean, standard error of the mean, 95% confidence intervals for differences of means, and *p* value. SAS Version 9.4 for Windows (SAS Institute, Cary, North Carolina) was used throughout.

## 3. Results

### 3.1. Plexin-B2 and Its Ligands Are Overexpressed in Majority of RMS Tissues and Correlated with Survival

**Figure 1:**
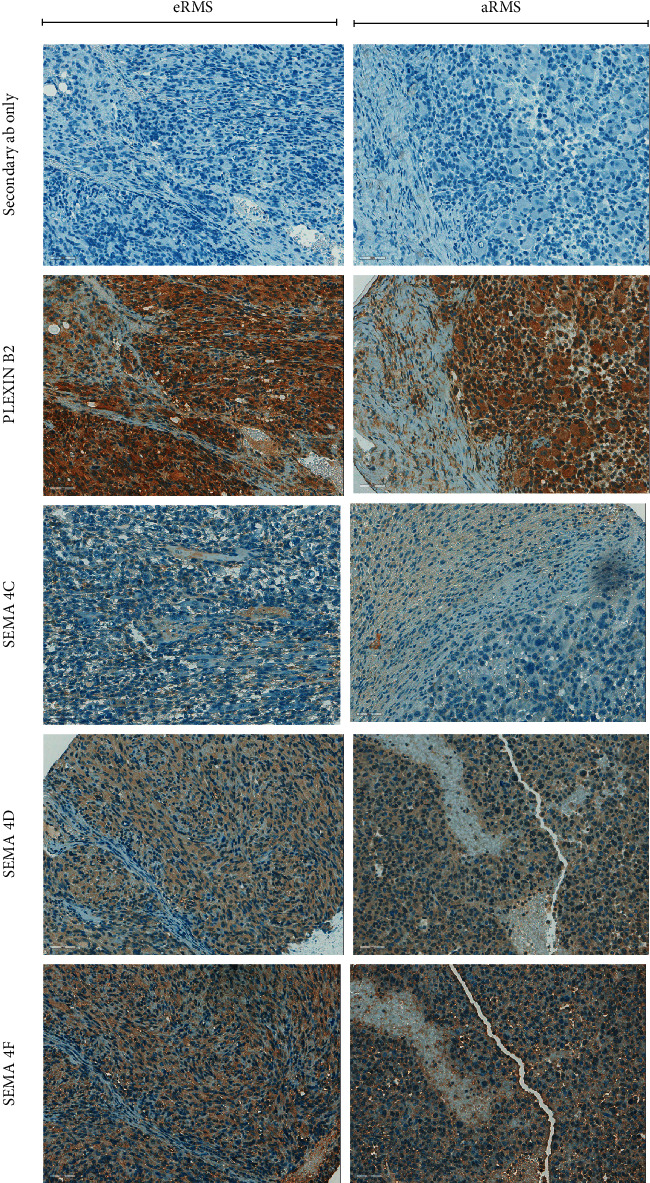
IHC of Plexin-B2 and its ligands in TMAs. Representative immunohistochemistry staining images showing the expression of Plexin-B2 and its ligands SEMA4C, SEMA4D, and SEMA4F in rhabdomyosarcoma subtypes (aRMS and eRMS). Secondary antibody for each antibody is used as a negative control. Scale bar: 50*μ*m.

To investigate protein expression of Plexin-B2, SEMA4C, SEMA4D, and SEMA4F, we performed IHC on formalin-fixed paraffin-embedded mouse and human RMS tissue microarray (TMA). Staining revealed that in both subtypes of RMS (eRMS and aRMS), the protein expressions of Plexin-B2 and its ligands SEMA4D and SEMA4F are elevated (see representative images in Figure 1). It is worthy of note that SEMA4D is the target of pepinemab (VX15/2503), recently investigated in pediatric phase I clinical trials (NCT03320330) ([18], [19]). IHC scores of Plexin-B2, SEMA4C, SEMA4D, and SEMA4 were calculated based on the percentage of stain present and the stain intensity of tumor cells in the tumor tissues of human patients and mouse models. Quantification of the stains in aRMS, aRMS, and undifferentiated pleiomorphic sarcomas (UPS) is summarized in Table 1. Overall, Plexin-B2 was present in 100% RMS cells in human tumor samples at low intensity in aRMS and at intermediate to high intensity in eRMS. In the mouse models tissues, Plexin-B2 expression was present in 75% of aRMS and 86% of eRMS tissues ranging from weak to strong staining intensity. The ligand SEMA4C was weakly expressed in 67% of human eRMS only. The SEMA4D and SEMA4F expression was seen in 100% of the human tumor cells, albeit at low intensity.

We next examined the clinical relevance of Plexin-B2 expression in RMS patients. The Kaplan–Meier curve indicated that high Plexin-B2 correlated with poorer overall survival in RMS patients in comparison to low Plexin-B2, with a statistical significance of 0.033 (Supplementary Figure S1). Overall, the data suggest that high protein and gene expression of Plexin-B2 have unfavorable survival outcomes in RMS patients.

### 3.2. Plexin-B2 Is Highly Expressed in RMS Cell Lines

**Figure 2:**
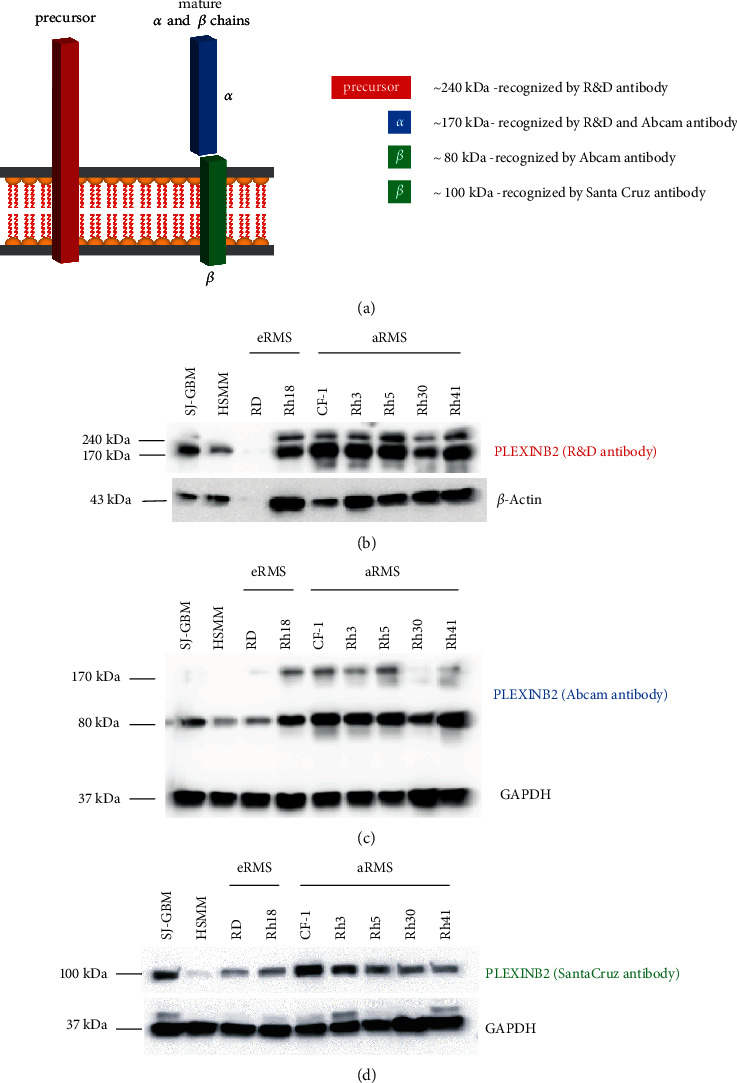
Validation of anti-Plexin-B2 antibodies to detect various forms of Plexin-B2 protein as well as detection of prominent Plexin-B2 expression in RMS cell lines. (a) Schematic representation of precursor and mature *a* and *ß* subunits of Plexin-B2 (adapted from Friedel, personal communication). (b) The antibody from the R&D Systems detects a precursor form (240 kDa) and a processed mature *a*-subunit (170 kDa). (c)The antibody from Abcam detects both processed mature *a* and *ß* subunits (170 kDa transmembrane and 80 kDa intracellular, respectively). (d) The Plexin-B2 antibody from Santa Cruz Biotechnology detects the transmembrane *ß*-subunit (85–100 kDa). Immunoblot analyses of protein lysates in various rhabdomyosarcoma cell lines, using Plexin-B2 antibodies from the R&D Systems, Abcam, and Santa Cruz Biotechnology. ß-Actin and GAPDH are used as loading controls. n.b. SJ-GBM and HSMM protein lysates served as controls.

Next, to confirm the protein expression of Plexin-B2, we performed immunoblot analyses using available cell culture models of RMS. To fully characterize isoform expression, we probed protein lysates of eRMS (RD and Rh18) and aRMS (Rh3, Rh5, Rh30, and Rh41) for Plexin-B2 using multiple antibodies which appropriately detect precursor and mature (*α* and *β*) forms of Plexin-B2 as illustrated in schematic Figure 2(a). Lysates from SJ-GBM (positive) and HSMM (negative) were used as controls for the western blots. Interestingly, all RMS cell lines harbored robust expression of Plexin-B2 subunits irrespective of the subtypes as depicted in Figures 2(b), 2(c), and 2(d) as detected by three independent Plexin-B2 antibodies. Notably, CF-1, a PDX explant primary tumor cell culture, showed the highest Plexin-B2 expression and RD (eRMS) showed the lowest Plexin-B2 expression compared to all the RMS cell lines. These results qualified the cell lines tested for further study of Plexin-B2 in rhabdomyosarcoma.

### 3.3. SEMA4C, SEMA4D, and SEMA4F Are Aberrantly Expressed in RMS Cell Lines

**Figure 3:**
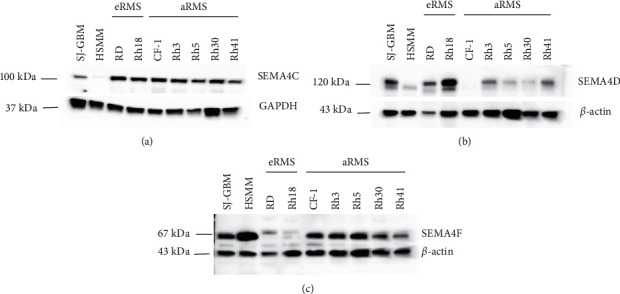
Robust expression of Plexin-B2 ligands. Immunoblot analyses of protein lysates of rhabdomyosarcoma cell lines, using the known ligands of Plexin-B2, i.e., SEMA4C, SEMA4D, and SEMA4F antibodies in panels (a), (b), and (c), respectively. SEMA4C is uniformly expressed between eRMS and aRMS cell lines. SEMA4D protein expression is increased in eRMS cell lines compared to aRMS cell lines. In contrast, SEMA4F protein expression is increased in aRMS cell lines compared to eRMS cell lines. GAPDH and ß-actin are used as loading controls.

To assess the expression pattern of the Plexin-B2 ligands, we probed the RMS protein lysates for SEMA4C, SEMA4D, and SEMA4F antibodies and detected membrane-bound SEMAs. In contrast to IHC quantification, SEMA4C expression was robust in all the tested RMS cell lines (eRMS and aRMS); SEMA4D expression pattern was aberrant between subtypes (Figures 3(a) and 3(b)). It is worthy of note that the eRMS cell lines, RD and Rh18, showed higher SEMA4D expression compared to aRMS cell lines (Rh3, Rh5, Rh30, and Rh41) and CF-1 showed the lowest SEMA4D expression among the tested cell lines. SEM4F showed a distinct expression pattern with low levels of SEMA4F in eRMS and high levels of SEM4F in aRMS cell lines. SEMA4D and SEMA4F displayed opposite expression trends between eRMS and aRMS cell lines (Figures 3(b) and 3(c)). These findings indicate that the ligand molecules of Plexin-B2 show distinct autocrine expression pattern in eRMS and aRMS. Specifically, SEMA4D expression is directly proportional to Plexin-B2 expression in the eRMS cell types. In contrast, SEMA4F expression was directly proportional to Plexin-B2 expression in the aRMS.

### 3.4. Efficient Knockdown of Plexin-B2 in RMS Cell Lines

**Figure 4:**
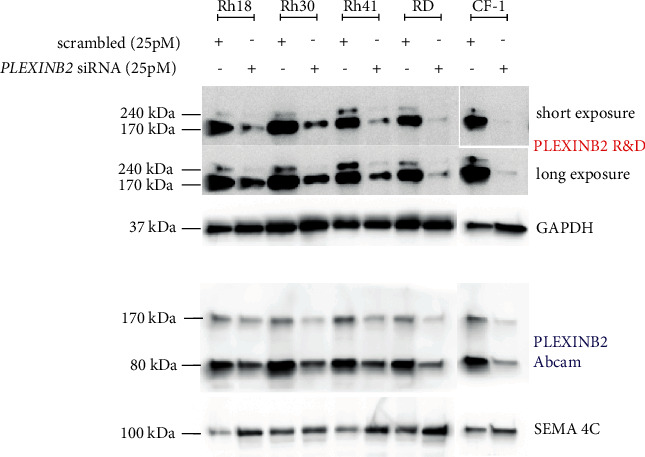
Efficient knockdown of Plexin-B2 siRNA in rhabdomyosarcoma. Plexin-B2 protein downregulation in selected eRMS and aRMS cell lines depicted by immunoblots. The cells were transfected with 25 picomoles of scrambled and Plexin-B2 siRNAs. Interestingly, the SEMA4C ligand expression is upregulated upon Plexin-B2 knockdown.

In order to determine the function of Plexin-B2 in cell growth and migration in RMS, we optimized knockdown of Plexin-B2 using two sets of siRNAs targeting Plexin-B2 and a set of non-targeting (scrambled) siRNA siControls. The siRNA-mediated knockdown of Plexin-B2 was conducted in two eRMS cell lines (Rh18 and RD) and three aRMS cell lines or cell cultures (CF-1, Rh30, and Rh41). Immunoblot analyses confirmed the efficient knockdown of Plexin-B2 in all the tested cell lines with both sets of siRNAs (siplexinB2_01 and siplexinB2_03) as depicted by representative immunoblots in Figure 4, Supplementary Figure S2, and Supplementary Figure S3. Notably, we found that SEMA4C expression increased after Plexin-B2 knockdown. In contrast, both SEMA4D and SEMA4F expressions remained unchanged upon Plexin-B2 knockdown. For the experiments that followed, we selected si_03 siRNAs as an efficient tool for Plexin-B2 knockdown.

### 3.5. Knockdown of Plexin-B2 Reduces Cell Proliferation in Cell Lines Rh30, Rh41, RD, and Rh18 but Not in CF-1

**Figure 5:**
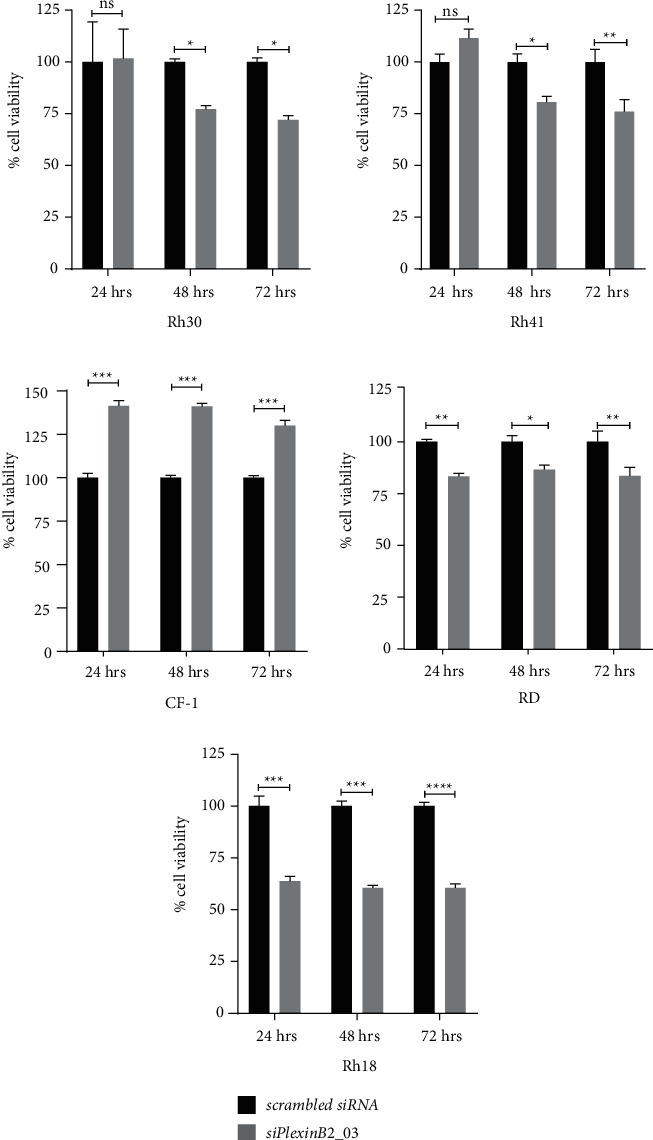
Effect of Plexin-B2 siRNA knockdown on cell proliferation. Cell growth assays were performed at 24 hr, 48 hr, and 72 hr after siRNA treatment in Rh30, Rh41, CF-1, RD, and Rh18. Plexin-B2 siRNA significantly reduced cell proliferation at 48 hr and 72 hr in Rh30 and Rh41 cell lines. Similarly, in eRMS cell lines, RD and Rh18, cell proliferation decreased significantly as early as 24 hr and persisted through 48 hr and 72 hr time points. However, in CF-1, cell proliferation significantly increased at all time points. (The data on graphs show means ± SEM. ^*∗*^*p* < 0.05, ^*∗∗*^ *p* < 0.01, ^*∗∗∗*^ *p* < 0.001).

To evaluate the effect of Plexin-B2 knockdown in cell proliferation, we selected three aRMS cell cultures (CF-1, Rh30, and Rh41) and two eRMS cell lines (Rh18 and RD) and measured cell viability at 24, 48, and 72 hr. Our results showed significant decrease in cell proliferation at 48 hr and 72 hr time points in aRMS cell lines, Rh30 and Rh41 (Figure 5). Cell growth after Plexin-B2 silencing was significantly decreased in RD and Rh18 cell lines at all time points. Plexin-B2 knockdown significantly (paradoxically) increased cell growth in CF-1. Overall, these results suggest a multifactorial and stochastic effect of Plexin-B2 knockdown on cell growth in RMS.

### 3.6. Neither Plexin-B2 Knockdown Nor Ectopic SEMA4C/SEMA4D Has Any Effect on Cell Migration in RMS Cell Lines

**Figure 6:**
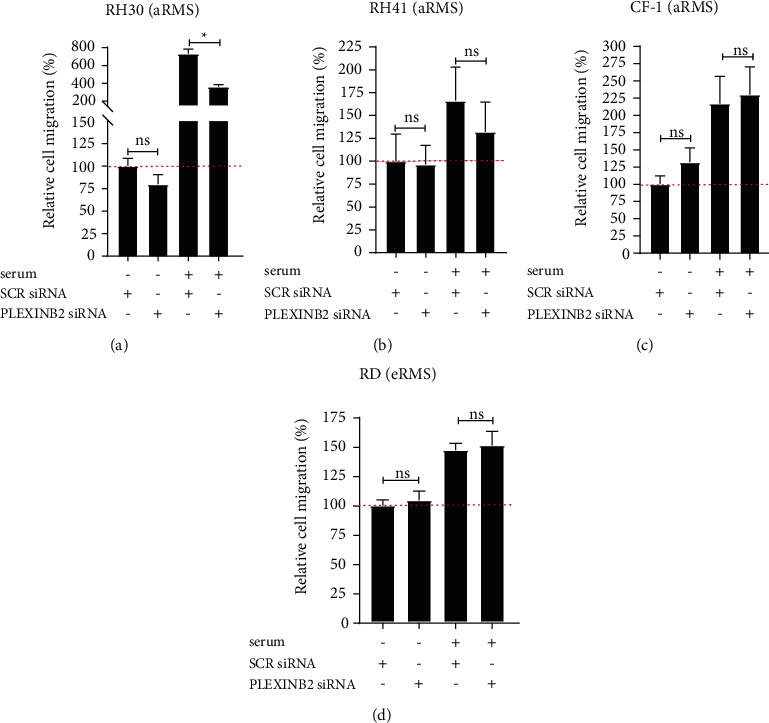
Effect of Plexin-B2 siRNA knockdown on cell migration. Crystal violet quantification of transwell migration and invasion assays reveals that in Rh30 and Rh41 (aRMS) cell lines, the migration and invasive properties are compromised upon Plexin-B2 knockdown in the context of serum bait. In contrast, migration property is increased in RD (eRMS) cell line. CF-1, a primary cell line, shows minimal effect on migration after Plexin-B2 knockdown. (The data on graphs show means ± SEM. ^*∗*^*p* < 0.05, ^*∗∗*^ *p* < 0.01, ^*∗∗∗*^ *p* < 0.001).

**Figure 7:**
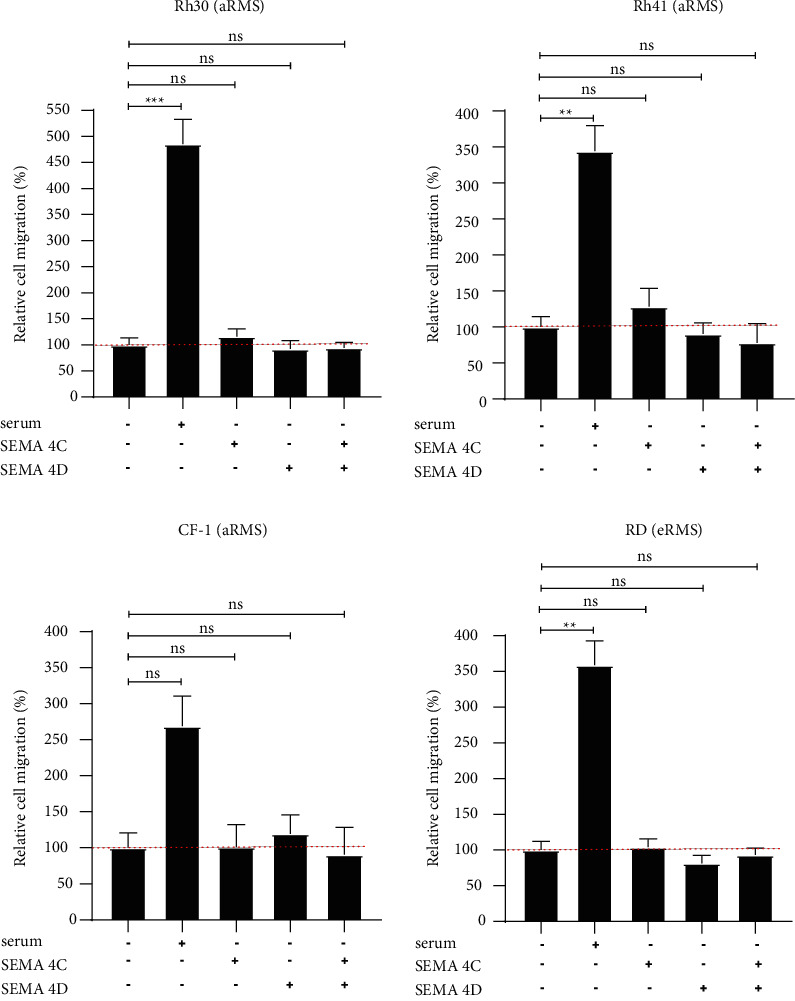
Effect of ligand stimulation in cell migration with and without serum. In a different xCELLigence instrument-based migration assay from the Boyden chamber assay in Figure 7, eRMS and aRMS cell lines have minimal response to ligand stimulation. SEMA4C and SEMA4D ligand concentration = 200 ng/mL. (The data on graphs show means ± SEM. ^*∗*^*p* < 0.05, ^*∗∗*^ *p* < 0.01. ^*∗∗∗*^ *p* < 0.001).

To determine whether Plexin-B2 regulates cell migration of RMS cell lines in ligand-independent or serum-responsive manner, we silenced Plexin-B2 using siRNAs in Rh30, Rh41, RD, and CF-1 (as shown in Figure 4 and Supplementary Figure S2) and then evaluated cell migration using transwell assays. The migrating cells were dyed with crystal violet, and the eluted dye intensity was quantified at 595 nm. The knockdown of Plexin-B2 caused a significant reduction in cell migration for only Rh30 (but not Rh41, CF-1, or RD) in the context of 10% fetal bovine serum bait (Figure 6(a)–6(d)) and Supplementary Figure S4). Next, we investigated whether the cognate ligands for Plexin-B2 stimulated migration of the Plexin-B2 expressing cell lines. Using a real-time conduction-based migration monitoring instrument (Figure 7), no change in migration was observed when ectopic SEMA4C or SEMA4D was used as a bait. Overall, these results revealed a multifactorial and stochastic effect of Plexin-B2 knockdown on cell growth in RMS, seemingly independent of SEMA4C or SEMA4D expression.

## 4. Discussion

In this study, we explored the role of Plexin-B2-SEMA4 signaling axis in the context of cell growth, cell migration, and invasion in RMS. We show that Plexin-B2 is highly expressed in aRMS and eRMS subtype at protein levels. From retrospective analysis of clinical IRSG-IV samples, high Plexin-B2 expression in new diagnosis samples correlated with poor patient survival in RMS. Furthermore, SEMA4C was robustly expressed in all the tested RMS cell lines, and SEMA4D was prominently expressed in eRMS cell lines. In contrast, SEMA4F was highly expressed in aRMS as compared to eRMS cell lines as revealed by immunoblot analyses. Knockdown of Plexin-B2 resulted in impaired cell growth in all immortalized RMS cell lines. However, the silencing of Plexin-B2 reduced serum-stimulated cell migration capacity in only one aRMS cell line and had no effect on any eRMS cell line; additionally, the Plexin-B2 cognate ligands SEMA4C and SEMA4D had no effect on migration in any of the Plexin-B2 expressing cell lines/cell cultures tested. A potential caveat of our cell viability assays is that CellTiter-Glo is a measure of cellular metabolism and therefore an indirect measure of cell viability and that viability and proliferation parameters generally but not always coincide.

Recent findings have reported that Plexin-B-SEMA4 signaling axis is overexpressed and involved in disease progression via cell proliferation and cell migration in bone, breast, and brain cancers [8, 9, 15–17, 20]. Related studies have revealed that SEMA4-Plexin-B signaling axis alters cell morphology and enhances cell migration and cell invasion by activating downstream signaling pathways such as Rho GTPases, RTK-Met, and AKT [8, 15, 17]. Our results indicate that Plexin-B2 can regulate cell migration in a selected cellular model of alveolar rhabdomyosarcoma. However, future experiments will be required for the assessment of noncanonical ligand dependence and downstream signaling pathways in RMS.

In summary, we have shown that Plexin-B2 can be a prognostic factor for RMS, implying that the Plexin-B-SEMA4 signaling axis might be a potential therapeutic target for rhabdomyosarcoma; however, paradoxical effects of Plexin-B2 knockdown to increase tumor cell growth and only selectively in certain cell lines to decrease tumor cell migration raise significant concerns about therapeutic approaches to Plexin-B-SEMA4 inhibition until the factors underlying this complex biology and clinical biomarkers of response can be developed.

## Figures and Tables

**Table 1:** IHC analyses of Plexin-B2, SEMA4C, SEMA4D, and SEMA4F using TMA for RMS.

	Plexin-B2 (*Homo sapiens*)					Plexin-B2 (*Mus musculus*)					
	aRMS		eRMS			aRMS		eRMS		UPS	
	# of samples	% of aRMS	# of samples	% of eRMS		# of samples	% of aRMS	# of samples	% of eRMS	# of samples	% of UPS
Stain present	1 of 1	100%	3 of 3	100%	Stain present	12 of 16	75%	6 of 7	86%	17 of 33	52%
% positive (tumor cells):					% positive (tumor cells):						
0	0	0%	0	0%	0	0	4%	0	0%	0	0%
1–10%	0	0%	0	0%	1–10%	0	0%	0	0%	0	0%
11–50%	0	0%	0	0%	11–50%	0	0%	0	0%	3	18%
51–80%	0	0%	0	0%	51–80%	0	0%	1	17%	1	6%
81–100%	1	100%	3	100%	81–100%	12	100%	5	83%	13	76%
Stain intensity (tumor cells):					Stain intensity (tumor cells):						
None	0	0%	0	0%	None	0	0%	0	0%	0	0%
Weak	1	100%	0	0%	Weak	1	8%	1	17%	5	29%
Intermediate	0	0%	1	33%	Intermediate	0	0%	0	0%	6	35%
Strong	0	0%	2	67%	Strong	11	92%	5	83%	6	35%

	SEMA4C (*Homo sapiens*)					SEMA4C (*Mus musculus*)					
	aRMS		eRMS			aRMS		eRMS		UPS	
	# of samples	% of aRMS	# of samples	% of eRMS		# of samples	% of aRMS	# of samples	% of eRMS	# of samples	% of UPS
Stain present	1 of 1	100%	3 of 3	100%	Stain present	13 of 16	81%	6 of 7	86%	20 of 33	61%
% positive (tumor cells):					% positive (tumor cells):						
0	1	100%	1	33%	0	1	8%	1	17%	5	25%
1–10%	0	0%	2	67%	1–10%	1	8%	1	17%	4	20%
11–50%	0	0%	0	0%	11–50%	7	54%	2	33%	7	35%
51–80%	0	0%	0	0%	51–80%	3	23%	1	17%	2	10%
81–100%	0	0%	0	0%	81–100%	1	8%	1	17%	2	10%
Stain intensity (tumor cells):					Stain intensity (tumor cells):						
None	1	100%	1	33%	None	1	21%	1	17%	5	25%
Weak	0	0%	2	67%	Weak	7	62%	4	67%	10	50%
Intermediate	0	0%	0	0%	Intermediate	4	14%	1	17%	1	5%
Strong	0	0%	0	0%	Strong	1	3%	0	0%	4	20%

	SEMA4D (*Homo sapiens*)					SEMA4D (*Mus musculus*)					
Stain present	1 of 1	100%	3 of 3	100%	Stain present	14 of 16	88%	6 of 7	86%	19 of 32	59%
	aRMS		eRMS			aRMS		eRMS		UPS	
	# of samples	% of aRMS	# of samples	% of eRMS		# of samples	% of aRMS	# of samples	% of eRMS	# of samples	% of UPS
% positive (tumor cells):					% positive (tumor cells):						
0	0	0%	0	0%	0	0	0%	0	0%	0	0%
1–10%	0	0%	0	0%	1–10%	0	0%	0	0%	1	5%
11–50%	0	0%	0	0%	11–50%	0	0%	0	0%	1	5%
51–80%	0	0%	0	0%	51–80%	2	14%	1	17%	3	16%
81–100%	1	100%	3	100%	81–100%	12	86%	5	83%	14	74%
Stain intensity (tumor cells):					Stain intensity (tumor cells):						
None	0	0%	0	0%	None	0	0%	0	0%	0	0%
Weak	1	100%	3	100%	Weak	8	57%	4	67%	11	58%
Intermediate	0	0%	0	0%	Intermediate	3	21%	2	33%	7	37%
Strong	0	0%	0	0%	Strong	3	21%	0	0%	1	5%

	SEMA4F (*Homo sapiens*)					SEMA4F (*Mus musculus*)					
	aRMS		eRMS			aRMS		aRMS		UPS	
	# of samples	% of aRMS	# of samples	% of eRMS		# of samples	% of aRMS	# of samples	% of eRMS	# of samples	% of UPS
Stain present	1 of 1	100%	3 of 3	100%	Stain present	12 of 16	75%	6 of 7	86%	18 of 33	55%
% positive (tumor cells):					% positive (tumor cells):						
0	0	0%	0	0%	0	0	0%	0	0%	0	0%
1–10%	0	0%	0	0%	1–10%	0	0%	0	0%	0	0%
11–50%	0	0%	0	0%	11–50%	0	0%	0	0%	1	6%
51–80%	0	0%	0	0%	51–80%	0	0%	0	0%	3	17%
81–100%	1	100%	3	100%	81–100%	12	100%	6	100%	14	78%
Stain intensity (tumor cells):					Stain intensity (tumor cells):						
None	0	0%	0	0%	None	0	0%	0	0%	0	0%
Weak	1	100%	1	33%	Weak	11	92%	5	83%	12	67%
Intermediate	0	0%	1	33%	Intermediate	1	8%	1	17%	5	28%
Strong	0	0%	1	33%	Strong	0	0%	0	0%	1	6%

## Data Availability

There are no extended datasets, and all data are presented as figures and tables in the manuscript.

## Supplementary Materials

Supplementary Materials Supplementary Table S1: STR cell profiling of the cell lines used in this study. Supplementary Figure S1: Plexin-B2, a prognostic factor. Kaplan–Meier survival curve shows that patients with high expression of Plexin-B2 have shorter survival. The data are adjusted for known survival covariates of stage, age, sex, histology, clinical group, site, and translocation status; *p*=0.033. Supplementary Figure S2: efficient knockdown of Plexin-B2 in Rh30, Rh41, and RD cell lines using two sets of siRNAs against Plexin-B2. Confirmation of Plexin-B2 knockdown in Rh30 (A, B), Rh41 (C, D), and RD (E, F) cell lines using antibodies from R&D which detects a precursor and alpha-chain subunit (240 kDa and 170 kDa, respectively) and Abcam which detects the intracellular alpha-subunit of 170 kDa and the transmembrane beta-chain subunit of 80 kDa. GAPDH is used as a loading control. n.b. Rh30 and Rh41 are alveolar rhabdomyosarcoma (aRMS) cell lines, and RD is an embryonal cell line (eRMS). Supplementary Figure S3: efficient knockdown of Plexin-B2 in Rh41, RD, and Rh30 cell lines using two sets of siRNAs at 25 picomole concentration against Plexin-B2. In this round, only siRNA_03 showed an efficient knockdown which is confirmed by Plexin-B2 immunoblot using R&D antibody. The SEMA4C levels were upregulated upon Plexin-B2 knockdown in RH41 and Rh30 cell lines. However, other ligands of Plexin-B2, SEMA4D, and SEMA4F levels were not altered when Plexin-B2 was absent. GAPDH is used as a loading control. n.b. Rh30 and Rh41 are alveolar rhabdomyosarcoma (aRMS) cell lines, and RD is an embryonal cell line (eRMS). Supplementary Figure S4: effect of Plexin-B2 siRNA knockdown on cell migration. Migration assays reveal that in Rh30 and Rh41 (aRMS) cell lines, the migration and invasive properties are compromised upon Plexin-B2 knockdown in the context of serum bait. In contrast, migration property is increased in RD (eRMS) cell line. CF-1, a primary cell line, shows minimal effect on migration after Plexin-B2 knockdown. (The data on graphs show means ± SEM. ^*∗*^*p* < 0.05, ^*∗∗*^*p* < 0.01, and ^*∗∗∗*^*p* < 0.001).
