# Supplementary material for: Plexin-B2 and Semaphorins Do Not Drive Rhabdomyosarcoma Proliferation or Migration
Source: Sarcoma. 2022 May 6;2022:9646909. doi: 10.1155/2022/9646909 (PMC9106520; doi:10.1155/2022/9646909)
Supplement: Supplementary Materials — Supplementary Table S1: STR cell profiling of the cell lines used in this study. Supplementary Figure S1: Plexin-B2, a prognostic factor. Kaplan–Meier survival curve shows that patients with high expression of Plexin-B2 have shorter survival. The data are adjusted for known survival covariates of stage, age, sex, histology, clinical group, site, and translocation status; p=0.033. Supplementary Figure S2: efficient knockdown of Plexin-B2 in Rh30, Rh41, and RD cell lines using two sets of siRNAs against Plexin-B2. Confirmation of Plexin-B2 knockdown in Rh30 (A, B), Rh41 (C, D), and RD (E, F) cell lines using antibodies from R&D which detects a precursor and alpha-chain subunit (240 kDa and 170 kDa, respectively) and Abcam which detects the intracellular alpha-subunit of 170 kDa and the transmembrane beta-chain subunit of 80 kDa. GAPDH is used as a loading control. n.b. Rh30 and Rh41 are alveolar rhabdomyosarcoma (aRMS) cell lines, and RD is an embryonal cell line (eRMS). Supplementary Figure S3: efficient knockdown of Plexin-B2 in Rh41, RD, and Rh30 cell lines using two sets of siRNAs at 25 picomole concentration against Plexin-B2. In this round, only siRNA_03 showed an efficient knockdown which is confirmed by Plexin-B2 immunoblot using R&D antibody. The SEMA4C levels were upregulated upon Plexin-B2 knockdown in RH41 and Rh30 cell lines. However, other ligands of Plexin-B2, SEMA4D, and SEMA4F levels were not altered when Plexin-B2 was absent. GAPDH is used as a loading control. n.b. Rh30 and Rh41 are alveolar rhabdomyosarcoma (aRMS) cell lines, and RD is an embryonal cell line (eRMS). Supplementary Figure S4: effect of Plexin-B2 siRNA knockdown on cell migration. Migration assays reveal that in Rh30 and Rh41 (aRMS) cell lines, the migration and invasive properties are compromised upon Plexin-B2 knockdown in the context of serum bait. In contrast, migration property is increased in RD (eRMS) cell line. CF-1, a primary cell line, shows minimal effect on migration after [file 9646909.f1.zip › 9646909.f1/Supplementary Table S1.docx]

**Supplementary Table S1. STR profiles of tumor cell cultures used**

| **Cell Culture** | **3S1358** |  | **TH01** |  | **D21S11** |  | **D18S51** | **Pe** | **nta_E** |  | **D5S818** | **D** | **13S317** |  | **D7S820** | **D** | **16S539** |  | **CSF1PO** | **Pe** | **nta_D** |  | **AMEL** |  | **vWA** | **D** | **8S1179** |  | **TPOX** |  | **FGA** |  |
| --- | --- | --- | --- | --- | --- | --- | --- | --- | --- | --- | --- | --- | --- | --- | --- | --- | --- | --- | --- | --- | --- | --- | --- | --- | --- | --- | --- | --- | --- | --- | --- | --- |
| CF-1-P15 | 17 | 17 | 9.3 | 9.3 | 29 | 30 | 14 | 14 | 7 | 12 | 11 | 13 | 11 | 12 | 10 | 11 | 11 | 11 | 10 | 12 | 10 | 12 | X | Y | 15 | 17 | 12 | 14 | 9 | 10 | 24 | 25 |
| RD-P33 | 15 | 17 | 9.3 | 9.3 | 28 | 29 | 13 | 18 | 12 | 12 | 11 | 11 | 13 | 13 | 8 | 12 | 10 | 11 | 10 | 10 | 11 | 13 | X | X | 18 | 18 | 11 | 15 | 9 | 9 | 20 | 21 |
| Rh18-P13 | 16 | 16 | 7 | 7 | 32.2 | 33.2 | 16 | 17 | 8 | 13 | 12 | 12 | 12 | 12 | 8 | 10 | 9 | 12 | 10 | 10 | 7 | 12 | X | X | 15 | 17 | 13 | 15 | 8 | 9 | 23 | 23 |
| Rh41-P85 | 17 | 17 | 7 | 9.3 | 29 | 31 | 15 | 16 | 11 | 17 | 10 | 13 | 8 | 9 | 10 | 11 | 12 | 13 | 11 | 12 | 9 | 12 | X | X | 16 | 18 | 10 | 13 | 8 | 11 | 20 | 22 |
| Rh30-P36 | 15 | 15 | 9 | 9.3 | 29 | 31.2 | 16 | 16 | 7 | 17 | 12 | 13 | 11 | 11 | 10 | 10 | 12 | 12 | 10 | 11 | 11 | 12 | X | Y | 17 | 18 | 12 | 15 | 8 | 11 | 22 | 22 |
| Rh5-p16 | 16 | 16 | 6 | 9.3 | 28 | 33.2 | 16 | 17 | 10 | 17 | 12 | 13 | 11 | 11 | 9 | 11 | 12 | 12 | 11 | 11 | 11 | 13 | X | Y | 16 | 19 | 12 | 13 | 8 | 11 | 21 | 21 |
