# Supplementary figures and images for: Plexin-B2 and Semaphorins Do Not Drive Rhabdomyosarcoma Proliferation or Migration
Source: Sarcoma. 2022 May 6;2022:9646909. doi: 10.1155/2022/9646909 (PMC9106520; doi:10.1155/2022/9646909)

Supplementary Figure S1

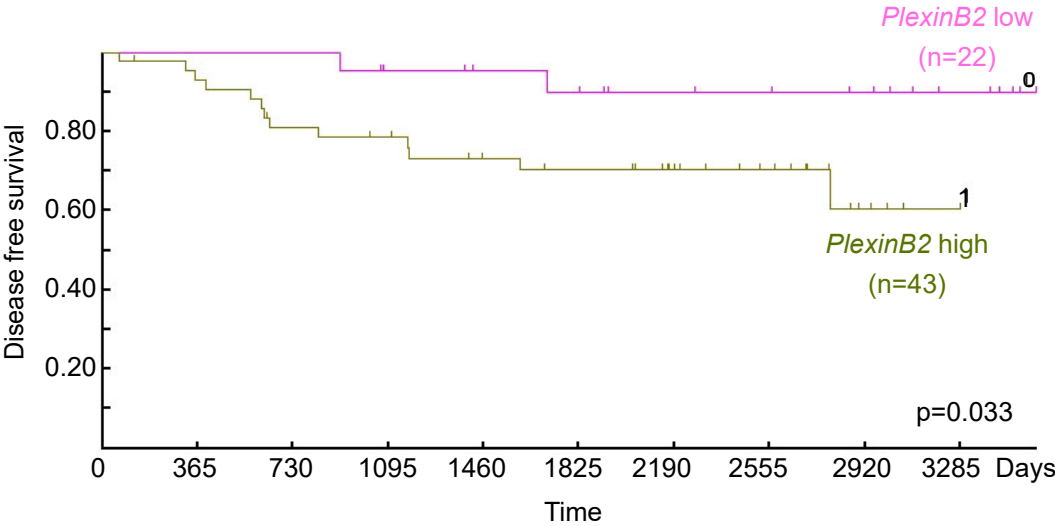

Supplement: Supplementary Materials — Supplementary Table S1: STR cell profiling of the cell lines used in this study. Supplementary Figure S1: Plexin-B2, a prognostic factor. Kaplan–Meier survival curve shows that patients with high expression of Plexin-B2 have shorter survival. The data are adjusted for known survival covariates of stage, age, sex, histology, clinical group, site, and translocation status; p=0.033. Supplementary Figure S2: efficient knockdown of Plexin-B2 in Rh30, Rh41, and RD cell lines using two sets of siRNAs against Plexin-B2. Confirmation of Plexin-B2 knockdown in Rh30 (A, B), Rh41 (C, D), and RD (E, F) cell lines using antibodies from R&D which detects a precursor and alpha-chain subunit (240 kDa and 170 kDa, respectively) and Abcam which detects the intracellular alpha-subunit of 170 kDa and the transmembrane beta-chain subunit of 80 kDa. GAPDH is used as a loading control. n.b. Rh30 and Rh41 are alveolar rhabdomyosarcoma (aRMS) cell lines, and RD is an embryonal cell line (eRMS). Supplementary Figure S3: efficient knockdown of Plexin-B2 in Rh41, RD, and Rh30 cell lines using two sets of siRNAs at 25 picomole concentration against Plexin-B2. In this round, only siRNA_03 showed an efficient knockdown which is confirmed by Plexin-B2 immunoblot using R&D antibody. The SEMA4C levels were upregulated upon Plexin-B2 knockdown in RH41 and Rh30 cell lines. However, other ligands of Plexin-B2, SEMA4D, and SEMA4F levels were not altered when Plexin-B2 was absent. GAPDH is used as a loading control. n.b. Rh30 and Rh41 are alveolar rhabdomyosarcoma (aRMS) cell lines, and RD is an embryonal cell line (eRMS). Supplementary Figure S4: effect of Plexin-B2 siRNA knockdown on cell migration. Migration assays reveal that in Rh30 and Rh41 (aRMS) cell lines, the migration and invasive properties are compromised upon Plexin-B2 knockdown in the context of serum bait. In contrast, migration property is increased in RD (eRMS) cell line. CF-1, a primary cell line, shows minimal effect on migration after [file 9646909.f1.zip › 9646909.f1/Supplementary Figure S1 .pdf]

# Supplementary Figure S2

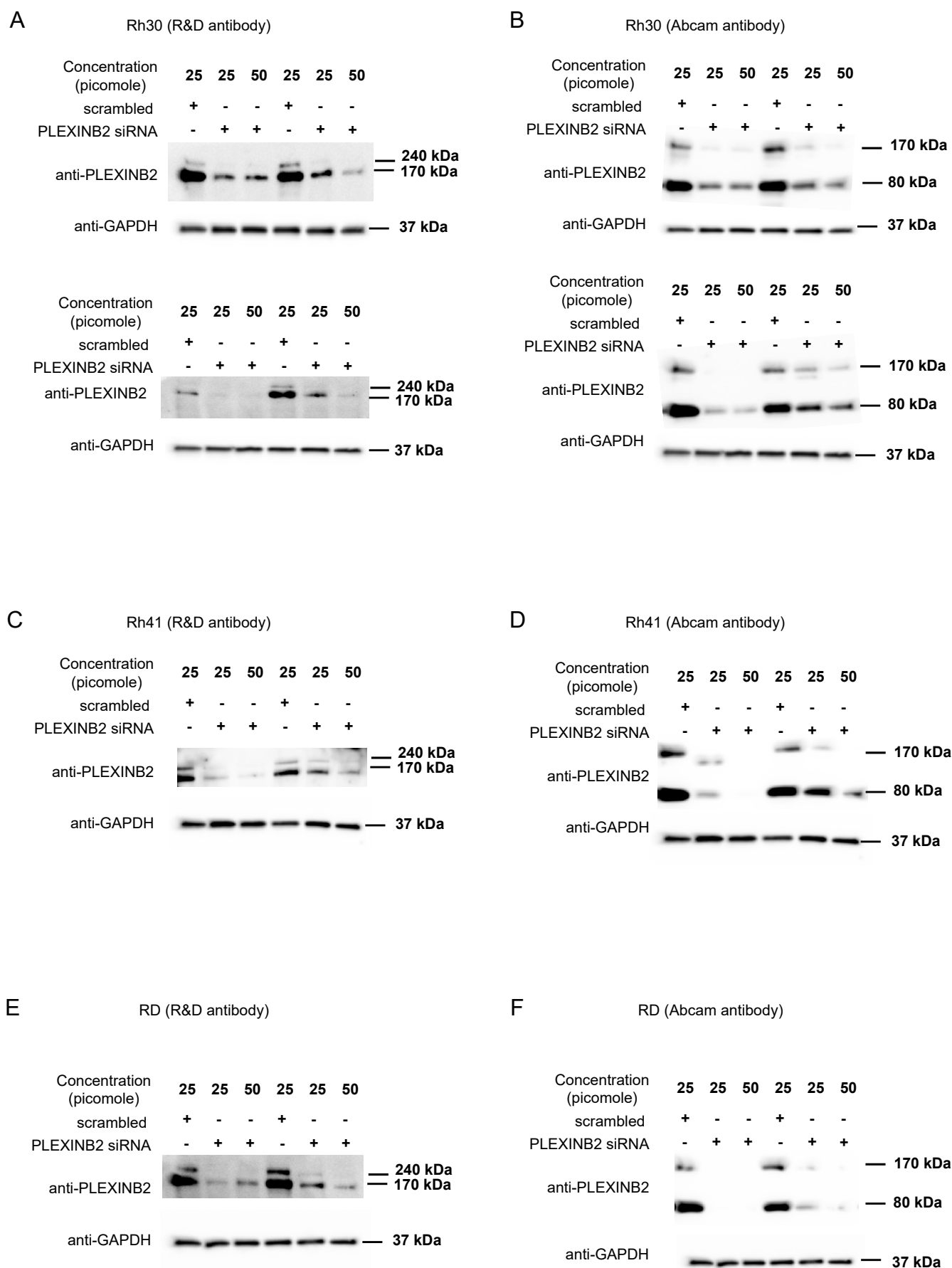

Supplement: Supplementary Materials — Supplementary Table S1: STR cell profiling of the cell lines used in this study. Supplementary Figure S1: Plexin-B2, a prognostic factor. Kaplan–Meier survival curve shows that patients with high expression of Plexin-B2 have shorter survival. The data are adjusted for known survival covariates of stage, age, sex, histology, clinical group, site, and translocation status; p=0.033. Supplementary Figure S2: efficient knockdown of Plexin-B2 in Rh30, Rh41, and RD cell lines using two sets of siRNAs against Plexin-B2. Confirmation of Plexin-B2 knockdown in Rh30 (A, B), Rh41 (C, D), and RD (E, F) cell lines using antibodies from R&D which detects a precursor and alpha-chain subunit (240 kDa and 170 kDa, respectively) and Abcam which detects the intracellular alpha-subunit of 170 kDa and the transmembrane beta-chain subunit of 80 kDa. GAPDH is used as a loading control. n.b. Rh30 and Rh41 are alveolar rhabdomyosarcoma (aRMS) cell lines, and RD is an embryonal cell line (eRMS). Supplementary Figure S3: efficient knockdown of Plexin-B2 in Rh41, RD, and Rh30 cell lines using two sets of siRNAs at 25 picomole concentration against Plexin-B2. In this round, only siRNA_03 showed an efficient knockdown which is confirmed by Plexin-B2 immunoblot using R&D antibody. The SEMA4C levels were upregulated upon Plexin-B2 knockdown in RH41 and Rh30 cell lines. However, other ligands of Plexin-B2, SEMA4D, and SEMA4F levels were not altered when Plexin-B2 was absent. GAPDH is used as a loading control. n.b. Rh30 and Rh41 are alveolar rhabdomyosarcoma (aRMS) cell lines, and RD is an embryonal cell line (eRMS). Supplementary Figure S4: effect of Plexin-B2 siRNA knockdown on cell migration. Migration assays reveal that in Rh30 and Rh41 (aRMS) cell lines, the migration and invasive properties are compromised upon Plexin-B2 knockdown in the context of serum bait. In contrast, migration property is increased in RD (eRMS) cell line. CF-1, a primary cell line, shows minimal effect on migration after [file 9646909.f1.zip › 9646909.f1/Supplementary Figure S2 .pdf]

Supplementary Figure S3

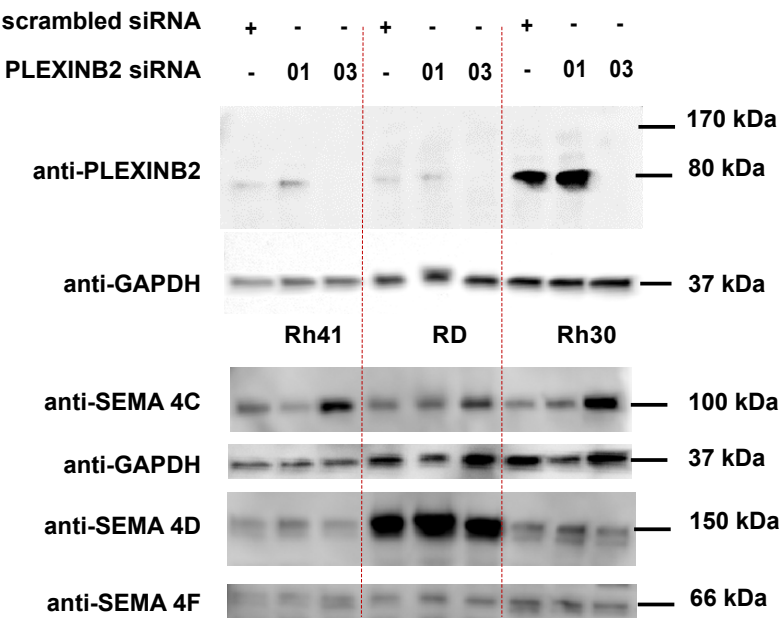

Supplement: Supplementary Materials — Supplementary Table S1: STR cell profiling of the cell lines used in this study. Supplementary Figure S1: Plexin-B2, a prognostic factor. Kaplan–Meier survival curve shows that patients with high expression of Plexin-B2 have shorter survival. The data are adjusted for known survival covariates of stage, age, sex, histology, clinical group, site, and translocation status; p=0.033. Supplementary Figure S2: efficient knockdown of Plexin-B2 in Rh30, Rh41, and RD cell lines using two sets of siRNAs against Plexin-B2. Confirmation of Plexin-B2 knockdown in Rh30 (A, B), Rh41 (C, D), and RD (E, F) cell lines using antibodies from R&D which detects a precursor and alpha-chain subunit (240 kDa and 170 kDa, respectively) and Abcam which detects the intracellular alpha-subunit of 170 kDa and the transmembrane beta-chain subunit of 80 kDa. GAPDH is used as a loading control. n.b. Rh30 and Rh41 are alveolar rhabdomyosarcoma (aRMS) cell lines, and RD is an embryonal cell line (eRMS). Supplementary Figure S3: efficient knockdown of Plexin-B2 in Rh41, RD, and Rh30 cell lines using two sets of siRNAs at 25 picomole concentration against Plexin-B2. In this round, only siRNA_03 showed an efficient knockdown which is confirmed by Plexin-B2 immunoblot using R&D antibody. The SEMA4C levels were upregulated upon Plexin-B2 knockdown in RH41 and Rh30 cell lines. However, other ligands of Plexin-B2, SEMA4D, and SEMA4F levels were not altered when Plexin-B2 was absent. GAPDH is used as a loading control. n.b. Rh30 and Rh41 are alveolar rhabdomyosarcoma (aRMS) cell lines, and RD is an embryonal cell line (eRMS). Supplementary Figure S4: effect of Plexin-B2 siRNA knockdown on cell migration. Migration assays reveal that in Rh30 and Rh41 (aRMS) cell lines, the migration and invasive properties are compromised upon Plexin-B2 knockdown in the context of serum bait. In contrast, migration property is increased in RD (eRMS) cell line. CF-1, a primary cell line, shows minimal effect on migration after [file 9646909.f1.zip › 9646909.f1/Supplementary Figure S3 .pdf]

Supplementary Figure S4

A RH30 (aRMS)

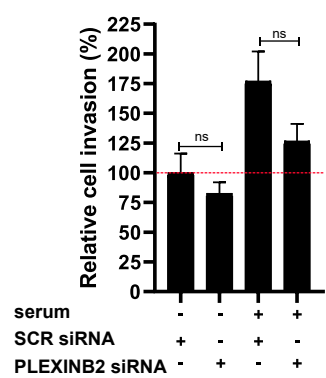

B RH41 (aRMS)

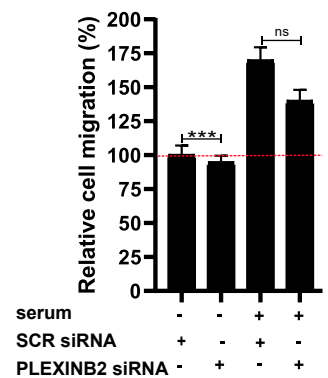

C CF-1 (aRMS)

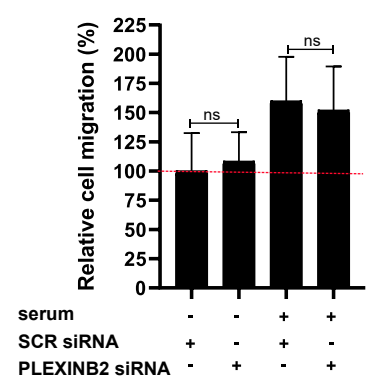

D RD (eRMS)

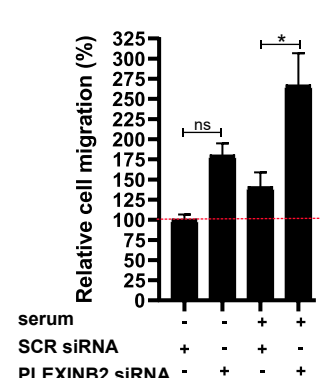

Supplement: Supplementary Materials — Supplementary Table S1: STR cell profiling of the cell lines used in this study. Supplementary Figure S1: Plexin-B2, a prognostic factor. Kaplan–Meier survival curve shows that patients with high expression of Plexin-B2 have shorter survival. The data are adjusted for known survival covariates of stage, age, sex, histology, clinical group, site, and translocation status; p=0.033. Supplementary Figure S2: efficient knockdown of Plexin-B2 in Rh30, Rh41, and RD cell lines using two sets of siRNAs against Plexin-B2. Confirmation of Plexin-B2 knockdown in Rh30 (A, B), Rh41 (C, D), and RD (E, F) cell lines using antibodies from R&D which detects a precursor and alpha-chain subunit (240 kDa and 170 kDa, respectively) and Abcam which detects the intracellular alpha-subunit of 170 kDa and the transmembrane beta-chain subunit of 80 kDa. GAPDH is used as a loading control. n.b. Rh30 and Rh41 are alveolar rhabdomyosarcoma (aRMS) cell lines, and RD is an embryonal cell line (eRMS). Supplementary Figure S3: efficient knockdown of Plexin-B2 in Rh41, RD, and Rh30 cell lines using two sets of siRNAs at 25 picomole concentration against Plexin-B2. In this round, only siRNA_03 showed an efficient knockdown which is confirmed by Plexin-B2 immunoblot using R&D antibody. The SEMA4C levels were upregulated upon Plexin-B2 knockdown in RH41 and Rh30 cell lines. However, other ligands of Plexin-B2, SEMA4D, and SEMA4F levels were not altered when Plexin-B2 was absent. GAPDH is used as a loading control. n.b. Rh30 and Rh41 are alveolar rhabdomyosarcoma (aRMS) cell lines, and RD is an embryonal cell line (eRMS). Supplementary Figure S4: effect of Plexin-B2 siRNA knockdown on cell migration. Migration assays reveal that in Rh30 and Rh41 (aRMS) cell lines, the migration and invasive properties are compromised upon Plexin-B2 knockdown in the context of serum bait. In contrast, migration property is increased in RD (eRMS) cell line. CF-1, a primary cell line, shows minimal effect on migration after [file 9646909.f1.zip › 9646909.f1/Supplementary Figure S4 .pdf]
